# Using RS and GIS for risk management of natural disasters consequences: The case of cultural heritage in Jinan city, China

**DOI:** 10.1016/j.heliyon.2024.e38217

**Published:** 2024-09-27

**Authors:** Guanyu Wei, Gab-Soo Han, Xiaoxia Lang

**Affiliations:** a Department of Environmental Landscape Architecture, College of Life Science, Gangneung-Wonju National University, Gangneung, 25457, South Korea; b Department of Environmental Design, College of Art and Design, Qingdao University of Technology, Qingdao, 266033, China

**Keywords:** Cultural heritage, Natural hazards, Risk map, GIS, AHP

## Abstract

The preservation of cultural heritage is confronted with significant challenges due to its extensive history and the increasing impact of climate change, particularly natural disasters. Instead of solely investing resources in post-disaster restoration efforts, implementing a proactive risk-management strategy for natural disasters is a more effective approach. This study introduces an analytical and evaluative methodology grounded in remote sensing (RS) and geographic information systems (GIS) to bridge the existing gap in understanding natural disaster risks to cultural heritage sites in Jinan, China. By leveraging a combination of RS data and established methodologies such as the Soil Conservation Service Curve Number, Maximum entropy, and the Revised Universal Soil Loss Equation, we conducted an in-depth analysis of the risks posed by various disasters including floods, landslides, earthquakes, and erosion. Furthermore, GIS and the Analytic Hierarchy Process were utilized to facilitate the risk assessment. Detailed disaster risk maps based on these assessments were produced. Our findings revealed that approximately 28.95 % of Jinan's cultural heritage sites face moderate to severe risks from natural disasters. Cultural heritage sites in Changqing District, Gangcheng District, and Laiwu District are particularly vulnerable to such calamities. These outcomes serve as crucial references for enhancing the safeguarding and management of cultural heritage, while informing disaster prevention and mitigation strategies in Jinan.

## 1 Introduction

Cultural heritage is a beacon of historical, artistic, and scientific significance that embodies the shared spiritual wealth of human civilization [1]. However, despite its profound value, cultural heritage remains susceptible to the ravages of natural disasters, which are often situated in exposed or semi-exposed environments. In 2020, statistics from the World Cultural Heritage Center of China revealed that 10.19 % of the country's cultural treasures suffered damage from such calamities [2]. According to a report from the State Administration of Cultural Heritage of China, more than 500 heritage sites were inundated by floods alone during the same year. As climate change intensifies, the frequency and severity of natural disasters increase, exacerbating the threat to cultural heritage sites. The increasing number of heritage sites imperiled by these environmental upheavals presents a formidable challenge to their preservation and protection [3,4].

Since the dawn of the 21st century, UNESCO has been increasingly vocal about the imperative to safeguard cultural heritage from the perils of disasters through a series of initiatives aimed at disaster risk reduction [5,6]. The 2005 World Conference on Disaster Reduction, convened by the United Nations General Assembly, brought the issue of cultural heritage to the forefront with the introduction of the Hyogo Framework for Action (HFA) 2005–2015. Subsequently, the 2008 World Heritage Periodic Report identified 14 primary threats to cultural heritage, including natural disasters such as floods, droughts, landslides, earthquakes, and erosion. In 2015, the Sendai Framework for Disaster Risk Reduction 2015–2030 was adopted as a follow-up to the HFA, incorporating the concept of “cultural heritage” into disaster resistance [7]. In alignment with this, the UNESCO World Heritage Center formulated a strategy for reducing risks from disasters at World Heritage Sites, prioritizing the identification, assessment, and monitoring of disaster risks in heritage areas. However, the comprehensive assessment of multiple natural disaster risks poses multifaceted challenges. This complexity arises from the need for intricate calculations to discern the factors that influence different disasters, compounded by variations in the interplay and significance of natural disasters across diverse regions [8]. Remote sensing (RS) and geographic information systems (GIS) are indispensable tools for streamlining model construction [9], indicator calculation, and data preprocessing in disaster risk assessment. Extensive research has been conducted on RS and GIS technologies to integrate natural disaster risk assessment into cultural heritage preservation efforts, yielding promising outcomes [[10], [11], [12]]. A methodological framework proposed by Agapiou et al. harnessed RS and GIS analysis, employing the Analytic Hierarchy Process (AHP) to assess disaster risks in the Paphos area of Cyprus [13]. Ravankhah et al. devised an assessment procedure to identify, analyze, and prioritize potential damage from natural disasters by applying it to the historic center of Rochemno in Greece [14]. Yagoub et al. utilized RS and GIS to evaluate the spatial distribution of natural disasters in the United Arab Emirates, map their proximity to heritage sites, and establish a comprehensive geographic information database for heritage preservation [15]. Khatakho et al. amalgamated AHP with GIS to produce a multi-hazard risk map of the Kathmandu Valley in Nepal, aiding disaster preparedness and mitigation efforts [16].

In China, extensive research has delved into the realm of natural disaster risks [17,18]; however, minimal attention has been directed towards assessing such risks in cultural heritage sites. Moreover, existing studies have predominantly revolved around singular hazard assessments [[19], [20], [21]], with only a few investigating the amalgamated risks posed by multiple hazards. Yang et al. utilized RS and GIS in conjunction with AHP to establish an assessment framework and facilitate the creation of a comprehensive risk map for fortified manors of Yongtai [22]. Zhao et al. focused on the Great Wall relics in Beijing and crafted natural disaster risk assessment models based on hazards and vulnerabilities [23]. Despite advancements made by these studies in elucidating the natural disaster risks faced by specific cultural heritage sites, a notable gap persists in conducting overarching analyses and research encompassing cultural heritage throughout the entire region.

This study proposes an innovative approach to comprehensively assessing the risk of natural disasters at cultural heritage sites using RS and GIS. To address this research gap in Jinan City, we utilized high-resolution RS satellite images to capture data on various factors influencing natural disasters in the region. Using multiple GIS-based methods, we identified areas prone to flooding, landslides, earthquakes, and erosion. Subsequently, the AHP was applied to evaluate the overall risk of natural disasters within a city from the perspectives of hazard and vulnerability. Detailed cultural heritage risk maps were generated, offering a comprehensive overview of the potential natural disaster risks facing Jinan's cultural heritage sites. This holistic perspective will facilitate the development of effective disaster prevention and mitigation strategies tailored to the challenges faced by Jinan. The findings presented here serve as a valuable resource for policymakers and stakeholders, informing them of proactive measures aimed at safeguarding the cultural heritage assets of Jinan against future disasters.

## 2 Study area

**Fig. 1:**
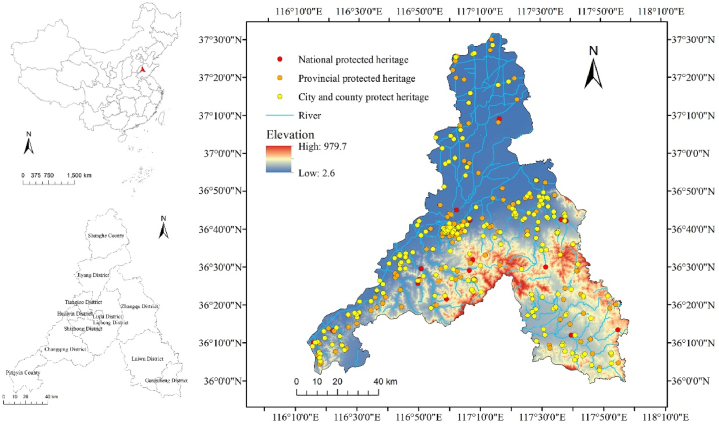
Map of the cultural heritage sites as designated at the national, provincial, and city and county levels in Jinan City. An inset map shows the location of Jinan within the administrative regions of China.

Jinan City is located in the central part of Shandong Province, China, between 36°01′–37°32′ N and 116°11′–117°59′ E, covering an area of approximately 10,236.7 km^2^ (Fig. 1). The topography of Jinan is high in the south and low in the north, with alluvial plains in the north and center, and mountainous areas and hills in the south. Jinan is characterized by typical continental monsoon climate, with an average annual temperature of 14.9 °C, and an annual average precipitation of 638.3 mm. Rainfall is mainly concentrated from June to September. The city is highly susceptible to natural disasters such as floods, landslides, and erosion due to short-term heavy rainfall. From 2010 to 2020, there were 36 torrential rains and floods in Jinan, and 27 landslides, mudslides, and other geological disasters were caused by Super Typhoon Lekima in August 2019 [24,25].

As one of the birthplaces of ancient Chinese civilization, Jinan boasts 437 cultural heritage sites registered with the Cultural Relics Bureau, ranking second in Shandong Province. In this study, a field investigation was conducted on Jinan's cultural heritage, with 449 cultural heritage sites obtained as study objects after sorting repeated and merged information on the sites (Table 1). Among them are 34 national, 178 provincial, and 237 city and county level cultural heritage sites, including ancient relics, tombs, architecture, grottos and stone carvings, as well as important historical sites and architecture in modern times.

**Table 1:** The added cultural heritage sites along with their sources.

The names of the added cultural heritage sites	The names registered by the Cultural Relics Bureau
The Western Changqing Section of the Qi Great Wall	Qi Great Wall relic
The Eastern Changqing Section of the Qi Great Wall	
The Licheng Section of the Qi Great Wall	
The Zhangqiu Section of the Qi Great Wall	
The Laiwu Section of the Qi Great Wall	
Bijia Mountain relic	Bijia Mountain, Qingyanggu Mountain fortress, and the Stone Wall ruins
Qingyanggu Mountain fortress	
The Stone Wall ruins	
Former Deutsche-Asiatische Bank building	The Weier Road modern architectural complex
Former German consulate	
Former postal administration building	
Former Bank of Communications building	
Former Minsheng Bank building	
Former German clinic	
Xiaoguanghan Cinema	

## 3 Materials and methods

### 3.1 Analytical framework

**Fig. 2:**
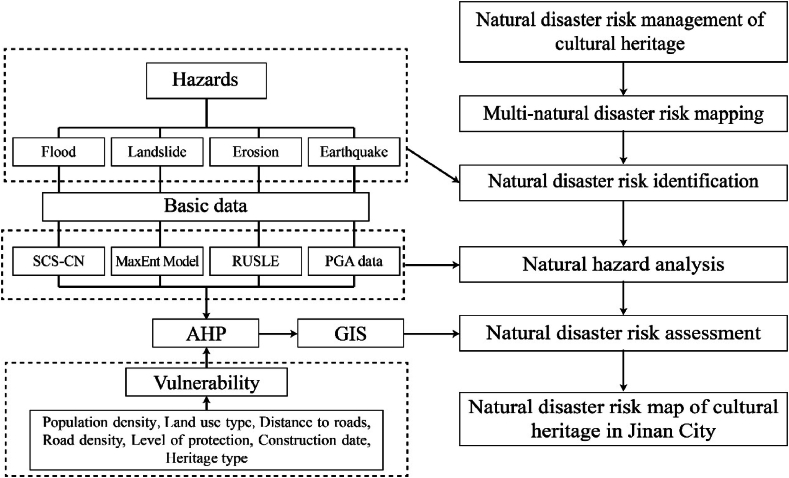
Analytical framework of the present study.

Currently, single disaster analyses and assessments are conducted based on hazards, exposure, and vulnerability [26,27]. However, because disasters may be induced by different environments and can cause varying subsequent effects, and because there are differences in evaluation indicators (e.g., exposure), a unified standard has not yet been formed for the comprehensive analysis and evaluation of multiple disasters. In general, relevant studies have been based on overlapping disasters or the interactions between multiple disasters [22,28]. The Managing Disaster Risks for World Heritage for World Heritage by UNESCO states that disaster risk is a product of hazards and vulnerability, referring to the chance of disaster happening that will impact cultural heritages [29]. This study builds on this risk assessment framework and evaluates the natural risks to cultural heritage sites in Jinan in three steps: identification, analysis, and assessment (Fig. 2). During the risk identification stage, floods, landslides, erosion, and earthquakes were selected as natural disaster risks. In the hazard analysis stage, a multi-hazard map illustrating the hazards of floods, erosion, and earthquakes in Jinan was drawn using GIS-based methods such as the Soil Conservation Service Curve Number (SCS-CN) and Revised Universal Soil Loss Equation (RUSLE), and a landslide hazard map of Jinan was drawn using the Maximum entropy (MaxEnt) model. Finally, in the risk assessment stage, a comprehensive assessment is performed using the evaluation index system constructed using the AHP based on the two dimensions of hazard and vulnerability.

### 3.2 Risk identification

According to the threat factors listed by UNESCO and research by national and international researchers, the assessment of natural disaster risks to cultural heritage mainly considers such factors as floods, landslides, erosion, earthquakes, salinization, and wildfires [13,30]. Jinan City is an inland city rather than a coastal one; therefore, salinization was not considered. The fire factor was not selected because of the difficulty in obtaining information on the spatial location of fires and the fact that no instances of fires at cultural heritage sites were recorded by the Jinan Municipal Cultural Relics Bureau. In summary, four types of natural disasters, namely floods, landslides, erosion, and earthquakes, were selected for this study and assessment.

### 3.3 Hazard analysis

After identifying the hazards, we collected the necessary RS and environmental data, proposed a GIS-based method to analyze multiple natural disasters in Jinan City, and drew disaster hazard maps.

#### 3.3.1 Flood hazard analysis method

The goal for estimating the flood risk is to evaluate the runoff volume for each watershed. Given the availability of data in the study area, we used a GIS-based SCS-CN model for the flood hazard analysis. This model, developed by the United States Department of Agriculture (USDA) in the 1950s, was used to estimate the runoff [31]. Compared with other commonly used hydrological models, it has the advantages of a simple structure and fewer required parameters; therefore, it is widely used to calculate runoff in small watersheds with insufficient hydrological data [32,33]. The model can be used to estimate the surface runoff based on the precipitation data of the watershed using the following equation.

(1) $$Q=\frac{{\left(P-I_{a}\right)}^{2}}{P+S-I_{a}}$$

where *Q* is the runoff depth (mm), *P* is the rainfall depth (mm), *S* is the potential maximum retention after the runoff begins (mm), and *I*_*a*_ is the initial abstraction (mm). Because empirical studies have found that *I*_*a*_ is highly related to *S* [34], *I*_*a*_ is typically defined using

(2) $$I_{a}=0.2S$$

Thus, the runoff equation can be approximately calculated using

(3) $$Q=\frac{{\left(P-0.2S\right)}^{2}}{P+0.8S}$$

Equation (3) shows that the runoff depth in the study area depends on the rainfall depth and the potential maximum retention after runoff begins. The potential maximum retention is related to the soil type, land use, and soil moisture conditions in the area [35]. The SCS model reflects the influence of these factors using a dimensionless parameter, the CN. Therefore, S can be calculated using the CN as follows:

(4) $$S=\frac{25400}{CN}-245$$

**Fig. 3:**
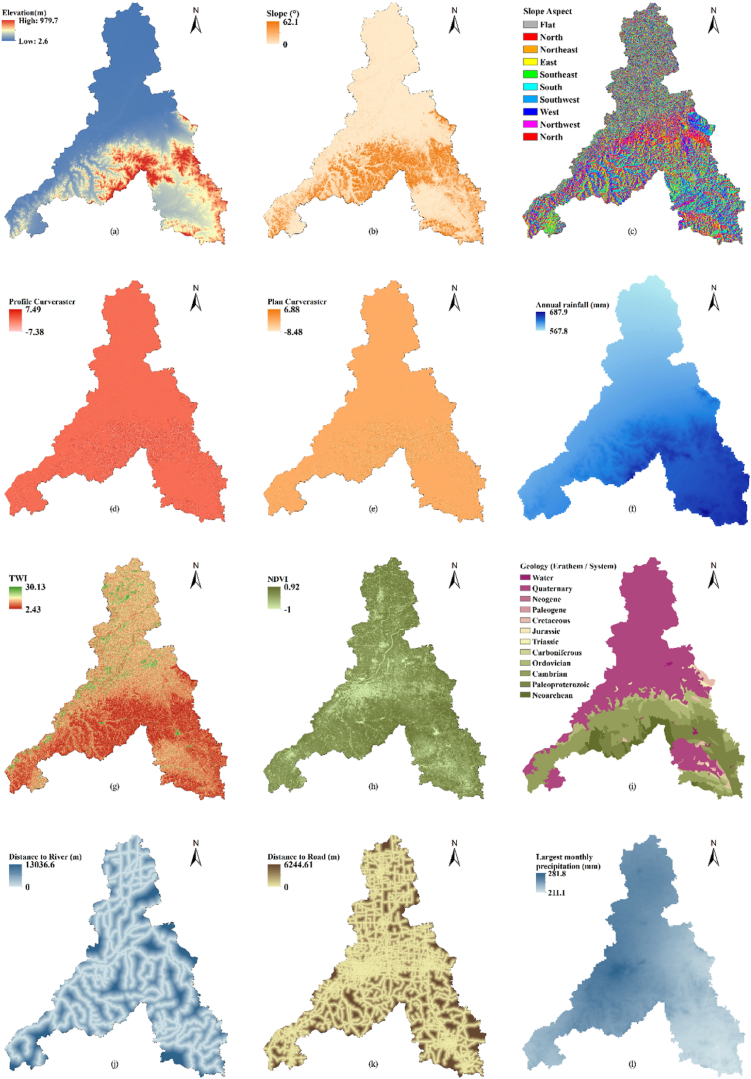

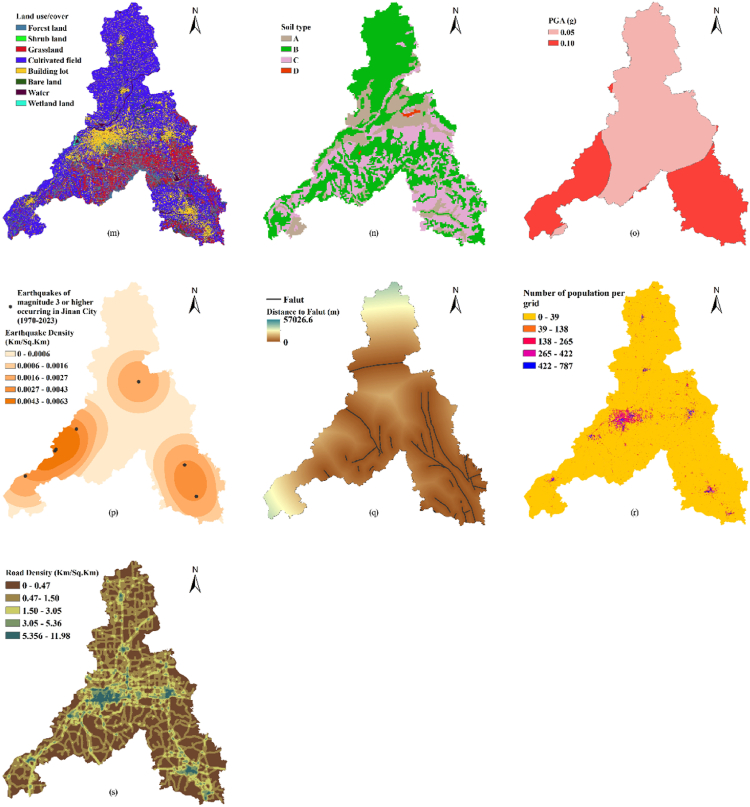
Thematic maps of Jinan City used in natural hazard analysis: (a) Elevation, (b) slope, (c) slope aspect, (d) profile curvature, (e) plan curvature, (f) Annual rainfall, (g) topographic wetness index (TWI), (h) NDVI, (i) geology, (j) distance to roads, (k) distance to rivers, (l) largest monthly precipitation, (m) land use/cover, (n) soil type, (o) PGA, (p) earthquake density, (q) distance to faults, (r) number of population per grid, (s) road density.

Based on the USDA proposed CN look-up table and empirical studies of CN values in China [36], we obtained the CN values for various land use types in Jinan City (Table 2). We also defined the soil types based on the four hydrological groups of soils introduced in Refs. [31,35], as shown in Table 2 (Fig. 3) (see Table 3).

**Table 2:** Runoff curve numbers for different soil types and land uses (antecedent moisture conditions II and Ia = 0.2 S).

Land use type	Soil type			
	A	B	C	D
Forest land	58	65	73	77
Shrub land	48	56	66	80
Grassland	45	60	66	78
Cultivated field	58	69	74	80
Building lot	70	81	88	92
Bare land	55	72	81	86
Water	95	95	95	95
Wetland	22	48	62	70

Notes: Antecedent moisture conditions II (Average Conditions): Represents typical or average soil moisture conditions, where the soil maintains a moderate moisture content and infiltration rate.

**Table 3:** The P-factor value for Jinan City.

Land use	Cultivated field	Shrub land	Forest land	Grassland	Building lot	Bare land	Water	Wetland
P-factor value	0.55	0.75	1	1	0	0	0	0

Soil type A includes soils with <10 % clay. Soil type B includes soils with 10–20 % clay. Soil type C includes soils with 20–40 % clay. Soil type D includes soils with >40 % clay.

Finally, the runoff volume of each watershed was calculated using equation (5), where *V*_*r*_ is the runoff volume in the watershed (m^3^), *Q* is the runoff depth (mm), and *A* is the watershed area (m^2^). Based on the assumption that rainfall runoff is retained within the watershed, potentially leading to submerged regions, iterative calculations are performed using the ArcGIS Surface Volume tool to correlate *V*_*r*_ with the surface volume across diverse elevations within the watershed [31]. When the *V*_*r*_ is close to the surface volume, the elevation represents the inundation threshold. Subsequently, the area below this elevation was extracted to identify the flood hazard areas.

(5) $$V_{r}=Q\times A/1000$$

#### 3.3.2 Landslide hazard analysis method

Landslide hazard was analyzed using the MaxEnt model. This model is a general-purpose machine learning-based model that is also based on information theory [37] and uses complete information to determine the (optimum) probability distribution of the objective [38]. Related studies have also confirmed that, compared with other landslide vulnerability prediction models, the Maxent model has the advantages of high computational efficiency, high prediction accuracy, and strong prediction ability [39]. We first assigned a space *X* to represent the geographic region of interest, which comprises a finite set of discrete grid cells. Each cell value represents the likelihood of a landslide occurring in that particular cell, with the sum of all cell values equal to 1. We also assigned a set of points m in *X*, each representing a site where a landslide was observed and recorded. Finally, by summarizing the results of previous research and considering the collected data [39,40], we selected data on height, slope, slope direction, profile curvature, plan curvature, annual rainfall, topographic wetness index (TWI), NDVI, geology, distance to rivers and roads to define the conditioning factors for *X* (Fig. 3a–k), with each grid cell x (x ∈ X) was assigned a non-negative probability value *P(x)* to represent the probability of landslide occurrence. To calculate *P(x)*, the function *f*_*x*_ was constructed to represent the information provided by the conditioning factors [41].

(6) $$f\left(x\right)=\lambda_{1}f_{1}+\lambda_{2}f_{2}+\lambda_{3}f_{3}+\lambda_{4}f_{4}+\cdots \cdots +\lambda_{i}f_{i}$$

where *λ*_*i*_ is a set of parameters and *f*_*i*_ is the value of the conditioning factor *i*.

The probability *P(x)* represents the distribution of the largest entropy. Pietra et al. [42] proved that the equation for the maximum entropy distribution can be written as:

(7) $$p\left(x\right)=\frac{{ef}_{x}\left(\cdots \right)}{Z}$$

where *Z* is a constant and *e* is the average of the function values *fx* in the landslide occurrence model and can be expressed as

(8) $$e=\frac{1}{m}\sum_{i=1}^{m}f_{xi}$$

The conditioning factor (*E*) of P must be close to *e*. The constraint condition (*lnZλ*) of *P* is *|e* − *E|* < *β*, where *β* is an arbitrary value.

(9) $$E=\sum_{x\in X}p\left(x\right)f_{x}$$

(10) $$InZ_{\lambda }=\frac{1}{m}\sum_{i=1}^{m}f_{xi}\left(\cdots \right)+\sum_{j}\beta_{j}\lambda$$

where *λ* is a set of parameters in the function *f*_*x*_.

In the MaxEnt software, we selected the predefined default values for tuning parameters that were 500 maximum iterations, 10,000 random background locations (pseudo-absence locations), an initial prevalence value of 0.5, a convergence threshold of 0.00001, and an automatic feature selection strategy.

#### 3.3.3 Erosion hazard analysis method

Different models have been developed to predict the erosion hazard, of which RUSLE is probably the most widely applied [43]. This model relies on information representing the major factors affecting erosion by water, that is, rainfall, soil texture, topography, and land cover and management, to quantify the mean annual erosion rates [44]. Its equation is:

(11) A=R∗K∗L∗S∗C∗P

where *A* is the mean annual soil loss, *R* is the rainfall factor, *K* is the soil erodibility factor, *L* is the slope steepness factor, *S* is the slope length factor, *C* is the land cover management factor and *P* is the support practice factor. To complete the *R*, *K*, *L*, *S*, and *C* factors [45,46], the following equations were applied:

(12) $$R=0.053p^{1.655}$$

where *R* represents the rainfall factor and *p* is the total annual precipitation in 2023 (mm).

(13) $$K=0.1317\times \left\{0.2+0.3\;\exp\left[-0.0265SAN\left(1-\frac{SIL}{100}\right)\right]\right\}\times {\left(\frac{SIL}{CLA+SIL}\right)}^{0.3}\times \left[1-\frac{0.25C}{C+\exp\left(3.72-2.95c\right)}\right]\times \left[1-\frac{0.7SN}{SN+\exp\;\left(22.9SN-5.51\right)}\right]$$

where *K* is the soil erodibility factor, *SAN* is the sand fraction content (%), *SIL* is the silt fraction content (%), *CLA* is the clay fraction content (%), and *C* is the organic carbon content (%); *SN=SAN/100*. The contents of sand, silt, clay, and organic carbon were obtained from soil type data in Shandong Province.

(14) $$\left\{ \begin{array}{c} L_{ij}=\frac{{\left(A_{i,j-in}+D^{2}\right)}^{m+1}-A_{i,j-in}^{m+1}}{D^{m+2}\times x_{i,j}^{m}\times 22.13^{m}}\\ m=\beta /\left(1-\beta \right)\\ \beta =\left(\sin\;\theta /0.0896\right)/[3.0{\left(\sin\;\theta \right)}^{0.8}+0.56] \end{array}\right.$$

where *L*_*i,j*_ is the impact of slope length factor for cell (*i,j*), *A*_*i,j−in*_ is the contributing area at the inlet of cell (*i,j*) measured in m^2^, *D* is the cell size in *m*, *X*_*i,j*_ = sin*α*_*i,*_j + cos*α*_*i,j*_*, α*_*i,j*_ is the aspect direction of cell (*i,j*), m is the ratio *β* of the rill to interill erosion, and *θ* is the slope angle in degrees.

(15) $$S=\left\{ \begin{array}{c} 10.8\;\sin\;\theta +0.03,\theta <5{}^{\circ}\\ 16.8\;\sin\;\theta -0.50,5{}^{\circ}\leq \theta \leq 10{}^{\circ}\\ 21.91\;\sin\;\theta -0.96,\theta \geq 10{}^{\circ} \end{array}\right.$$

where *S* is the slope length factor and *θ* is the slope angle in degrees.

(16) $$C=\left\{ \begin{array}{c} 1,c=0\\ 0.6508-0.3436\;\ln\;c,0<c\leq 78.3\%\\ 0,c>78.3\% \end{array}\right.$$

(17) $$c=\frac{NDVI-{NDVI}_{\min}}{{NDVI}_{\max}-{NDVI}_{\min}}$$

where C is the land cover management factor, c is the vegetation coverage, and NDVI_max_ and NDVI_min_ are the values of the normalized difference vegetation index (NDVI) at 95 % and 5 % cumulative probability levels, respectively.

The P-factor was obtained by assigning values to lands with different land-use types based on the literature experience [47,48], after an overlap analysis was performed using ArcGIS on the current land-use data and slope data in Jinan City.

#### 3.3.4 Earthquake hazard analysis method

Owing to the scarcity of reliable and detailed geological and geotechnical engineering data for Jinan, we conducted an earthquake hazard analysis based on data from the China Earthquake Peak Ground Acceleration (PGA) Zoning Map (GB18306-2015), which was released by the China Earthquake Administration and completed based on China's probabilistic earthquake hazard analysis [49]. Such an analysis considers the spatial and temporal heterogeneity of seismic activities in China, and the resulting PGA values are more consistent with the characteristics of seismic activities in China.

### 3.4 Risk evaluation

Using the AHP method, this study proposes an indicator system based on the two dimensions of hazard and vulnerability to evaluate the natural disaster risk to cultural heritage sites in Jinan City. The concept of hazard involves the probability of disaster occurrence, that is the acquired risk level for each natural disaster. Vulnerability refers to the susceptibility or exposure of cultural property to hazards. It depends on the location or specific characteristics of the cultural heritage [29], including population density, land-use type, distance to roads, road density, level of protection, construction date, and heritage type.

According to statistics, Jinan witnessed an average annual number of rainstorm days (daily rainfall >50 mm) of up to 16.3, the most frequent type of natural disaster in the city. Therefore, we believe that floods pose the greatest potential threat with the highest weight. Landslide disasters, which are caused by heavy rainfall, are second only to floods. Earthquake disasters, despite having the strongest destructive effect on cultural heritage, have less significance than flood and landslide disasters because Jinan has a relatively stable geological structure, registering a low earthquake magnitude in history [25]. Erosion causes relatively little harm to cultural heritage and thus has the lowest significance [22]. Standardized values for each factor were then calculated according to the risk level.

The exposure level of cultural heritage sites was measured by population density, land-use type, distance to roads, and road density. Population density and land use are the most direct indicators of the social environment. Regions with high population densities and urbanization rates can lead to environmental changes that affect the stability of cultural heritage sites. Furthermore, attention and funding for heritage conservation may be diverted by housing and infrastructure development, and lead to the transfer of funds from cultural heritage protection to urban disaster prevention and relief. Although the continuous development and extension of road transportation networks render cultural heritage sites more accessible, the environment, within and outside these sites, is affected. Large bridges and high transportation capacities can affect the structure and foundation of adjacent cultural heritage sites, with a dense road network spoiling the ecological landscape of heritage sites [50]. The distance to roads and road density was derived by performing Euclidean distance and line density analysis on road data in ArcGIS and then applying the Jenks Natural Breaks Classification Method to divide them into 5 classes.

The specific characteristic of cultural heritage was measured by the level of protection, construction date, and heritage type. The level of protection represents the risk management ability of the cultural heritage sites. The *Law of the People's Republic of China on Protection of Cultural Relics* establishes that immovable cultural heritage sites should be safeguarded at the national, provincial, city and county levels based on their historical, artistic, and scientific significance [51]. However, local governments generally prioritize the protection of cultural heritage at the national level over that at a lower level. Therefore, this study selected protection level as an evaluation indicator. The status quo of cultural heritage is associated with the date and type of construction [27]. Based on data from the third national survey of cultural relics, we categorized the construction dates into five groups: the Qin dynasty (before 207 BC), Tang dynasty (907), Yuan dynasty (1368), and Qing dynasty (1912). Different types of cultural heritage adopt distinct materials and thus have varying abilities to withstand natural disasters. Compared to grottos and stone carvings built with stones, bricks, or concrete and the important historical sites and architecture of modern times, relics and ancient architecture made of soil and wood are more susceptible to natural disasters [27].

In this study, the Yaahp software was used to construct the judgment matrices for hazard and vulnerability, resulting in the weights for each element. The scoring of the pair-wise comparison in each matrix was done by the co-authors. The min-max standardization method was used to convert all values into a standardized range of 0–1, with the weights and standardized scores in Table 4 used to calculate the hazard and vulnerability scores for each cultural heritage site. The natural disaster risk to cultural heritage sites was calculated as follows:

(18) $$H=\sum_{i=1}^{n}H_{ij}\times W_{i}$$

(19) $$V=\sum_{i=1}^{n}V_{ij}\times W_{i}$$

(20) R=H×V

where H is the hazard score and V is the vulnerability score. *n* represents the number of factors, *H*_*ij*_ denotes the score of the *j* classification factor in the *i* hazard factor, *V*_*ij*_ represents the *j* classification factor in the *i* vulnerability factor, and *W*_*i*_ is the weight of the *i* evaluation factor. A higher R value indicates a higher risk of natural disasters.

**Table 4:** Quantification standards of hazard and vulnerability indices.

Dimension	Index	Abbr.	Weight	Type	Score	Standardized Score
Hazard	Flood	H1	0.490	Non-flooded area	1	0
				100-year flood/storm	2	0.25
				50-year flood/storm	3	0.5
				20-year flood/storm	4	0.75
				10-year flood/storm	5	1
	Landslide	H2	0.305	Very low	1	0
				Low	2	0.25
				Medium	3	0.5
				High	4	0.75
				Very high	5	1
	Earthquake	H3	0.126	Very low	1	0
				Low	2	0.5
				Medium	3	1
	Erosion	H4	0.079	Slight erosion	1	0
				Mild erosion	2	0.2
				Moderate erosion	3	0.4
				Intense erosion	4	0.6
				Extremely intense erosion	5	0.8
				Violent erosion	6	1
Vulnerability	Population density	V1	0.196	Very low	1	0
				Low	2	0.25
				Medium	3	0.5
				High	4	0.75
				Very high	5	1
	Land-use type	V2	0.160	Forest land	1	0
				Shrub land	1	0
				Grassland	1	0
				Cultivated field	4	0.75
				Building lot	5	1
				Bare land	2	0.25
				Water	5	1
				Wetland	3	0.5
	Distance to roads (m)	V3	0.072	>4683.46	1	0
				3434.54–4683.46	2	0.25
				1873.38–3434.54	3	0.5
				624.46–1873.38	4	0.75
				<624.46	5	1
	Road density	V4	0.072	Very low	1	0
				Low	2	0.25
				Medium	3	0.5
				High	4	0.75
				Very high	5	1
	Level of protection	V5	0.167	National level	1	0
				Provincial level	2	0.5
				City and county level	3	1
	Construction date	V6	0.167	Before the Qin dynasty	5	1
				From the Qin to Tang dynasties	4	0.75
				During the Song and Yuan dynasties	3	0.5
				During the Ming and Qing dynasties	2	0.25
				After 1912	1	0
	Heritage type	V7	0.166	Relic	5	1
				Ancient tomb	3	0.5
				Grotto, stone carving	2	0.25
				Ancient architecture	4	0.75
				Important historical site and architecture in modern times	1	0

### 3.5 Data collection

The main data sources used in this study are listed in Table 5. Data on the location, type, protection level, and construction date of each cultural heritage site were obtained from the Jinan Cultural Relics Bureau. Google Earth images from January 2024 were used as a reference to reconfirm the spatial location, latitude and longitude of the cultural heritage sites. Natural environment data were mainly obtained from the Resource and Environment Science and Data Center, the Chinese Academy of Sciences, and the National Geological Data Center. These data were preprocessed before further analysis and evaluation (Fig. 3).

**Table 5:** Source of basic data in the study area.

Data name	Resolution	Data sources
Cultural heritage list of Jinan City	–	Jinan Cultural Relics Bureau (http://jnwl.jinan.gov.cn/)
Landsat8 OLI data (2023)	30m	USGS (http://glovis.usgs.gov/)
Digital elevation model data of Jinan City	30m	Resource and Environment Science and Data Center, Chinese Academy of Sciences (https://www.resdc.cn/)
Water system data of Jinan City	–	Resource and Environment Science and Data Center, Chinese Academy of Sciences (https://www.resdc.cn/)
Monthly precipitation dataset for China from 1901 to 2021	1 km	National Tibetan Plateau Data Center (https://data.tpdc.ac.cn/)
Annual precipitation data for Jinan City from 2023	1 km	China Meteorological Data Service Center (https://data.cma.cn/)
Data of landslide points in Jinan City over the years	–	Geographic remote sensing ecological network platform (ht tp://www.gisrs.cn)
Data of earthquake points in Jinan City over the years	–	National Earthquake Data Center (https://data.earthquake.cn/)
PGA date of Jinan City	–	China Earthquake Peak Ground Acceleration Map (https://www.gb18306.net)
Geological data of Shandong Province	1 km	National Geological Data Center (http://dc.ngac.org.cn/Home)
Fault data of Shandong Province	–	National Geological Data Center (http://dc.ngac.org.cn/Home)
Soil type data of Shandong Province (2020)	1 km	National Soil Information Service Platform of China (http://www.soilinfo.cn/)
NDVI	30m	Resource and Environment Science and Data Center, Chinese Academy of Sciences (https://www.resdc.cn/)
Administrative division data, road data	30m	Shuijingzhu omnipotent map Downloader
The spatial distribution of population in 2020, China	100m	WorldPop (https://www.worldpop.org/)

## 4 Results

### 4.1 Natural hazard zones in jinan

#### 4.1.1 Floods

To analyze the flood hazard in Jinan, we used the SCS-CN method to simulate flood-prone areas with different return periods of floods, thus presenting the flood hazard areas in the city, including those subjected to 10-, 20-, 50-, and 100-year floods. First, the digital elevation model data of Jinan were processed using the hydrology toolbox in ArcGIS, with rivers in the city divided into 20 watersheds. Based on relevant studies and historical meteorological data, it was concluded that rainfall depths for different return periods of floods were correlated with monthly rainfall [31]. Therefore, we expressed the rainfall depths for 10-, 20-, 50-, and 100-year floods as 25 % (70.45 mm), 50 % (140.9 mm), 100 % (281.8 mm), and 150 % (422.7 mm), respectively, of the largest monthly precipitation during 1901–2022. Then, data on precipitation rate, land-use type, and soil type were used to calculate the runoff depths for each watershed using equations (3), (4), and the runoff volume for each watershed was calculated using equation (5). Finally, substitution calculations were performed in GIS using the Surface Volume tool to obtain a flood hazard map for Jinan.

**Fig. 4:**
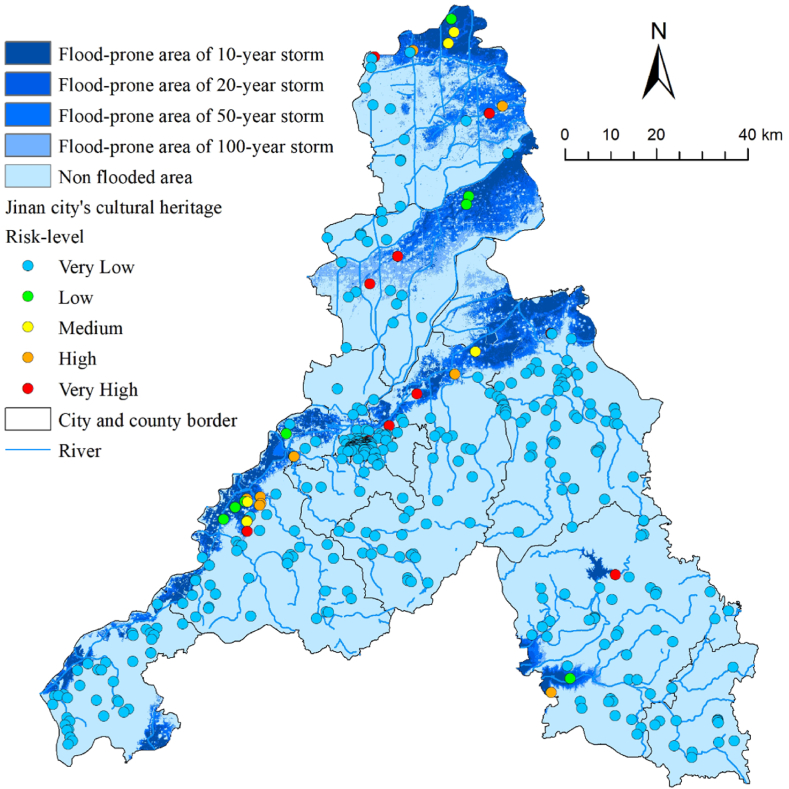
Flood hazard classification and cultural heritage flood risk-level map of Jinan City.

As shown in Fig. 4, although Jinan is prone to heavy rainfall in summer, it has a relatively high percentage of areas with a very low flood hazard because of the geographic advantage of a dense river network. The area of the extremely high-hazard regions was 572.73 km^2^, accounting for 5.59 %, and was mainly distributed along the lower reaches of the rivers in the central and northern regions (Fig. 10). However, because the riverbed of the Yellow River in the central region of Jinan is 3–4 m higher than the ground on both sides, there are no obvious high-hazard areas along the banks. Eight cultural heritage sites were located in very high-hazard areas (1.78 %), most of which were relics before the Song and Yuan dynasties. This type of cultural heritage mostly served as settlement sites for ancient humans who first selected areas that were close to water sources and suitable for cultivation as construction sites.

#### 4.1.2 Landslides

**Fig. 5:**
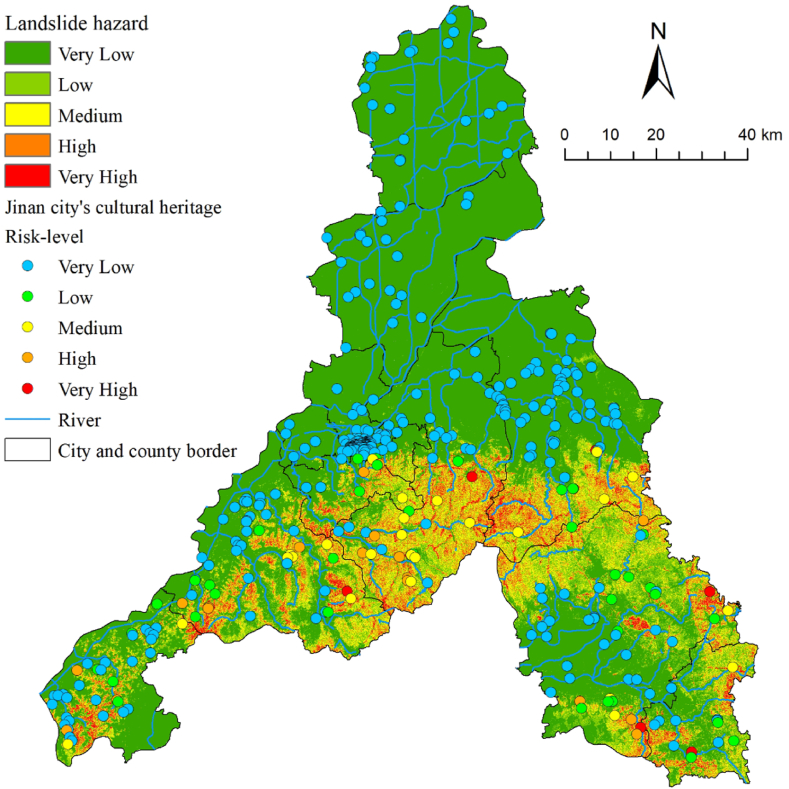
Landslide hazard classification and cultural heritage landslide risk-level map of Jinan City.

**Fig. 6:**
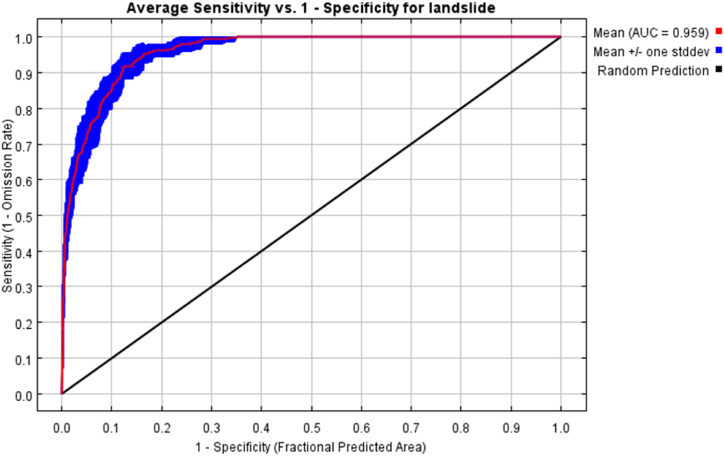
The AUC-ROC of the validation dataset using MaxEnt models.

Maxent 3.4.4 software was used to analyze landslide hazard in Jinan. The Maxent model typically requires two datasets, including the 11 conditioning factors mentioned earlier and a dataset of 42 historical landslides in Jinan City. The datasets were in a 30 m grid format. The specific operation was to import the two datasets processed in GIS into the Maxent model, taking 75 % of the landslide data as the training set and 25 % of the data as the test set, which was used to train the data and build the model. The test set was used to validate the reliability of the model. After running the model 10 times, the average of the results of the 10 runs was taken as the final evaluation result. Finally, the acquired results were categorized into five classes (very high, high, medium, low, and very low) using Natural Breaks in GIS to obtain a landslide hazard map for Jinan (Fig. 5). Notably, for the prediction results of the Maxent model, an accuracy test should be conducted using the receiver operating characteristic (ROC) curve; the closer the area under the ROC curve (AUC) value is to 1, the higher the model prediction accuracy. Fig. 6 shows the AUC-ROC curve obtained after 10 runs. The obtained AUC value reached 0.959, indicating excellent prediction accuracy.

Very high-hazard landslide areas in Jinan, with an area of 243.7 km^2^ (2.4 %), are mainly located in the southern part of the city, concentrating in Taishan Mountain with an elevation of 200m and a slope of over 20°. Despite having a high vegetative cover, this area features a low TWI and high hydraulic gradient, and road construction-induced excavation of the slope foot and the influence of the river on the banks lead to an increased hazard of landslides. Although there are a small number of cultural heritage sites located in very high-hazard regions (six), all are in southern mountainous areas, which makes it difficult for the government to conduct monitoring activities; thus, targeted protection is required.

#### 4.1.3 Erosion

**Fig. 7:**
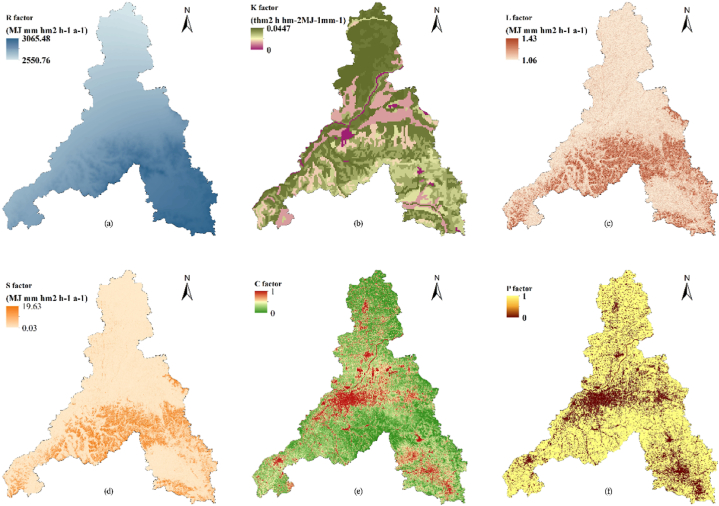
Thematic maps of Jinan City used in erosion hazard analysis: (a) R-factor, (b) K-factor, (c) L-factor, (d) S-factor, (e) C-factor, (f) P-factor.

**Fig. 8:**
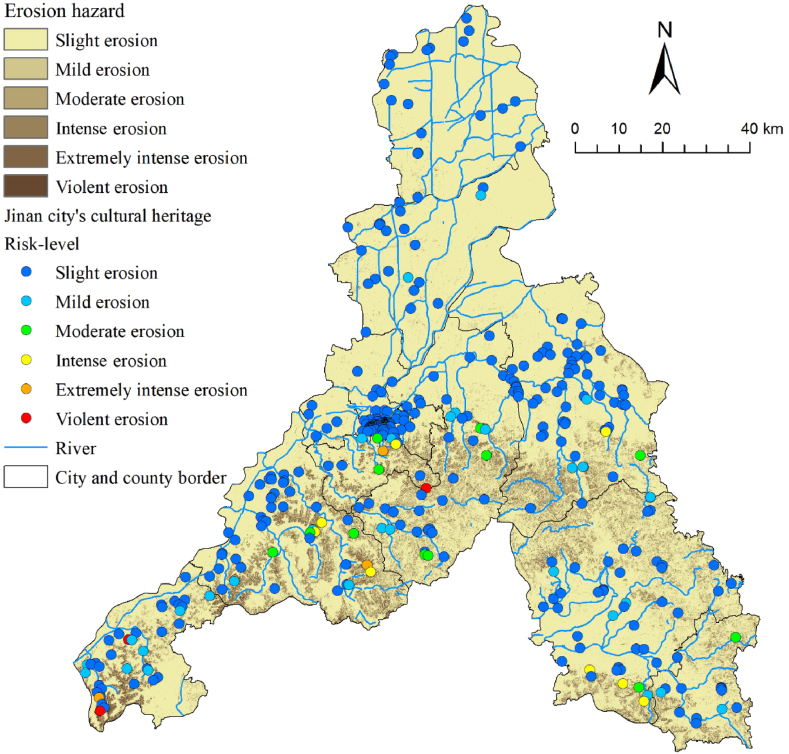
Erosion hazard classification and cultural heritage erosion risk-level map of Jinan City.

To determine the erosion of Jinan, we calculated the six types of factors in equation (11) separately (Fig. 7) and finally obtained the average erosion modulus of Jinan as 804.8 t/km^2^.a, and the annual erosion amount was 823.8t. According to the *Standards for Classification and Gradation of Soil Erosion* (SL109-2007) issued by the Ministry of Water Resources of China (Table 6), the erosion intensity of Jinan was classified using an erosion hazard map (Fig. 8).

**Table 6:** Standards for classification and gradation of soil erosion in China.

Gradation standards (t/km^2^-.a)	<200	200–2500	2500–5000	5000–8000	8000–15000	>15000
Level	Slight erosion	Mild erosion	Moderate erosion	Intense erosion	Extremely intense erosion	Violent erosion

Hydraulic erosion represented the main erosion type in Jinan City, a process where water causes the removal and transportation of soil, rock, or sediment from one location to another. Unlike other types of erosion, hydraulic erosion occurs through the action of flowing water, such as rivers, rainfall, and glacial meltwater. In Jinan, slight erosion accounted for up to 80.1 %; intensity or higher levels of erosion accounted for 4.9 % (Fig. 10) and were mainly concentrated in the southern area of Taishan Mountain. Three cultural heritage sites, which are located in an area of violent erosion, are distributed on the top or cliffs of the southern Taishan Mountain, with steep terrain, large slopes, and fast water currents, making them susceptible to soil washing. Two of these are national cultural heritage sites that require a high level of protection. Therefore, appropriate measures should be taken as soon as possible.

#### 4.1.4 earthquake

**Fig. 9:**
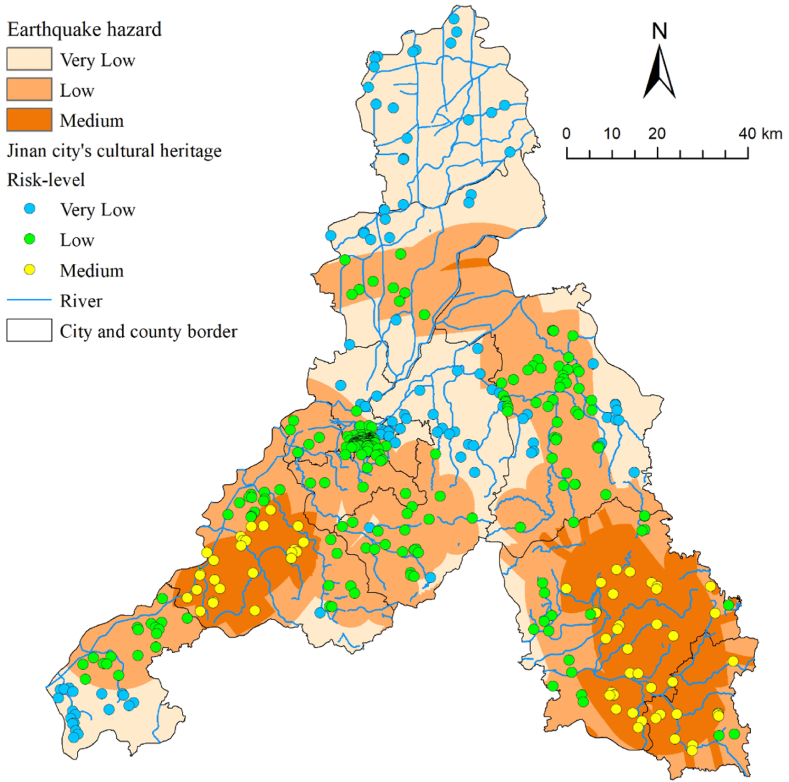
Earthquake hazard classification and cultural heritage earthquake risk-level map of Jinan City.

A PGA map of Jinan was obtained by cropping a PGA shake map of China along the boundary of the study area. Fig. 3 shows that the overall PGA of Jinan is ≤ 0.1 g. In previous studies, such a value was considered a “low hazard,” and thus not at significant hazard [52]. However, considering the potential damage to cultural heritage caused by small earthquakes, we consulted the literature [53], obtained data on earthquakes of magnitude 3 or higher occurring in Jinan City (1970–2023), and data on faults. Finally, we generated earthquake density and distance to faults from these datasets and overlaid these data in ArcGIS to conduct a seismic hazard analysis (Fig. 3p and q). Since Jinan is not located in China's seismic hazard regions and lacks the geological background for strong earthquakes [54], and according to the Jinan Earthquake Monitoring Center, only three earthquakes of magnitude 3.0 or higher have occurred since 2006 (http://jneq.jinan.gov.cn/), the earthquake risk is considered low. Therefore, we no longer established high-hazard areas for the acquired results (Fig. 9).

**Fig. 10:**
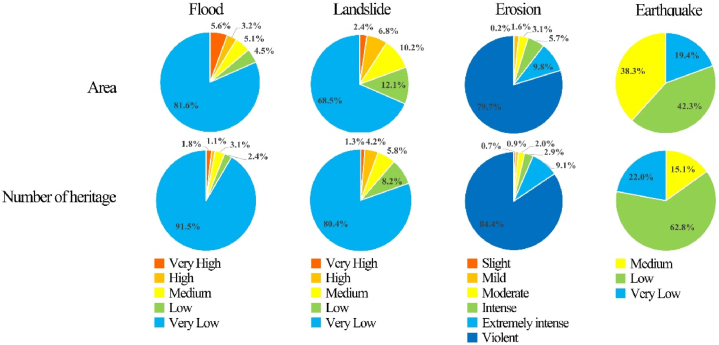
Comparison of the percentage of hazard areas for various types of natural disasters and the number of cultural heritage sites.

The very low, low, and medium hazard regions in Jinan cover an area of 3719.9 km^2^, 4532.5 km^2^, and 1984.4 km^2^, respectively (Fig. 10). The Spatial Join tool in ArcGIS was used to analyze earthquake hazards to cultural heritage sites in Jinan City, of which 68 were located in medium-hazard areas. Most cultural heritage sites are old, with relatively low levels of building materials and techniques. For these sites, precautionary measures should be taken after on-the-spot investigations. These measures may include modern and traditional seismic reinforcements and regular monitoring and maintenance of structures.

### 4.2 Analysis of the natural disaster risk assessment results

**Fig. 11:**
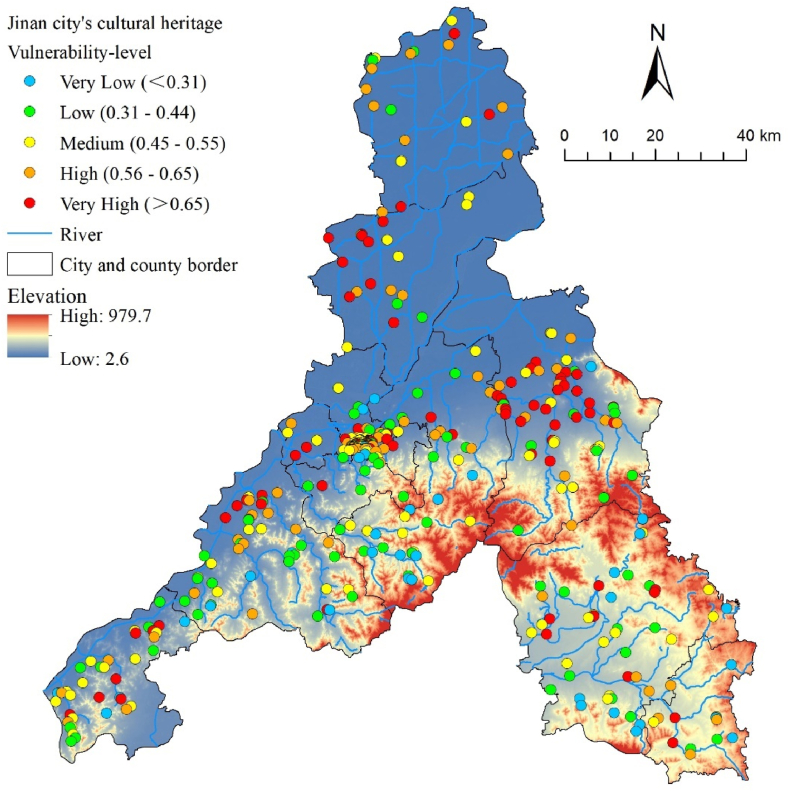
Cultural heritage vulnerability map of Jinan City.

**Fig. 12:**
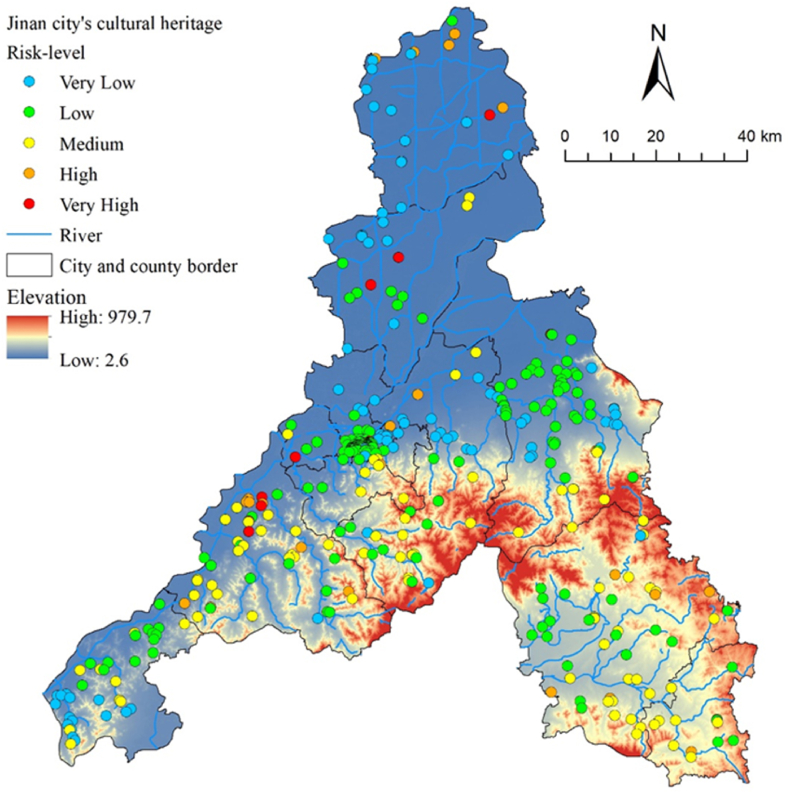
Natural disaster risk classification map of Jinan City.

The above four types of natural disaster analysis results and vulnerability indicators were taken in sequence and calculated according to the weights to obtain H, V (Fig. 11), and R values using a Field Calculator in ArcGIS, to draw a natural disaster risk map for cultural heritage sites. Subsequently, the risks were classified into five categories based on Natural Breaks: very low, low, medium, high, and very high (Fig. 12).

The results showed an average R-value of 0.058 for cultural heritage in Jinan. As shown in Table 7, the percentage of cultural heritage sites with very high risk is 2.45 %. These sites were mainly ancient cultural heritage sites from the pre-Qin dynasty. Because humans gave priority to settling in areas close to water sources during this period, these heritage sites are all distributed along the lower reaches of rivers, at low altitudes, and are extremely vulnerable to floods. High-risk cultural heritage sites account for 6.9 %, mainly located in the North Dasha River and Mouwen River Basins, the upper reaches of which are at a higher altitude, with great terrain undulation and high rainfall, subject to landslides and soil erosion due to river erosion and rainwash, whereas the lower reaches are susceptible to flooding. In addition, faults with a PGA value of 1.0 are distributed in these areas, and attention should be paid to the potential risk of earthquake disasters.

**Table 7:** Natural disaster risk assessment and classification results of Jinan City.

Level	Value interval	Number of heritages	Percentage
Very Low	0–0.020	83	18.49 %
Low	0.021–0.064	236	52.56 %
Medium	0.065–0.136	88	19.60 %
High	0.137–0.237	31	6.90 %
Very High	>0.238	11	2.45 %

**Fig. 13:**
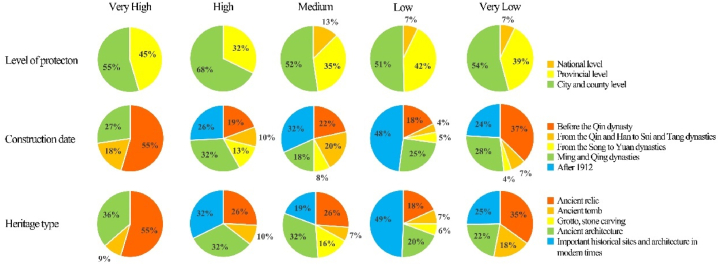
Fan chart grouped by level of protection, construction date, and heritage type.

**Fig. 14:**
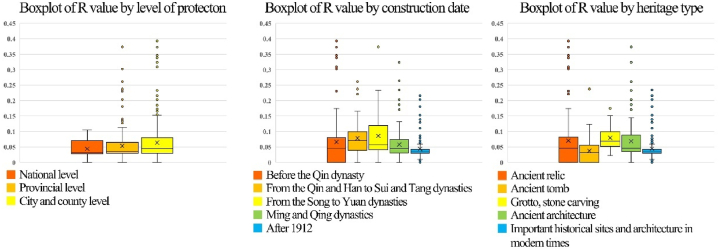
Boxplots of R-value grouped by level of protection, construction date, and heritage type.

The results also show that cultural heritage sites at the city and county levels are exposed to more severe natural hazard risks. Those sites facing high and very high risks, 9 (82 %) and 24 (71 %), respectively account for the highest proportion (Fig. 13). In the R-value boxplots divided by protection level (Fig. 14), the median R-value of cultural heritage at the city and county levels is 0.063, which is markedly higher than that of national (0.049) and provincial (0.048) cultural heritage. Among cultural heritage sites with very high risks, those constructed before the Qin dynasty accounted for the highest proportion, according to the analysis results of the year of construction for cultural heritage. However, the R-value results show that natural disaster risk is higher for cultural heritage from the Qin to Tang dynasties, with a median of 0.076, followed by that of the Song and Yuan Dynasties (0.073). Regarding the types of cultural heritage sites, those involving grottos, and stone carvings had the highest R values, although they were small in number. Moreover, many relics and ancient architecture are at high or above risk.

## 5 Discussion

### 5.1 A natural disaster risks comprehensive assessment method

Analyzing and evaluating natural disaster risks to cultural heritage sites is an important part of cultural heritage protection and management. Many studies have drawn disaster risk maps based on the AHP method [22,55], which is an element of subjectivity. This study used the SCS-CN method, MaxEnt model, RUSLE method, and a PGA map of China to analyze the natural disaster hazards in Jinan City. According to the vulnerability indicator of cultural heritage, a natural disaster risk map for cultural heritage in Jinan was drawn, thereby facilitating the prediction of the potential risks caused by natural disasters to cultural heritage sites. This method can be used to provide an accurate and efficient preliminary assessment of natural disaster risk for cultural heritage sites with limited data and can be a useful reference for making decisions about the protection of cultural heritages and identifying cultural heritages that need a more detailed analysis.

Unlike previous studies that determine disaster risk areas by weighted superposition of historical disaster information or geographical data, we utilized proven and relatively effective disaster analysis models and calculation methods, rendering natural hazard analysis more objective and scientific. The resulting hazard maps are not limited to cultural heritage and can also be integrated with social and economic indicators, applying to urban critical infrastructure, such as social infrastructure, energy, and business infrastructure. Additionally, this study can identify specific cultural heritage sites in high-risk areas, assist the heritage site managers or owners with reliable information on individual or overall risk levels, and enable them to take effective preventive measures.

This study attempts to construct a comprehensive evaluation index system for various natural disaster risks based on the dimensions of hazard and vulnerability. Owing to the limited data available, assessing the exposure and vulnerability of cultural heritage sites remains a challenge. Previous studies typically determined exposure based on the natural and social environments of heritage sites [50,56]. However, considering that different natural disaster risk areas in Jinan have diverse natural environments (for instance, high-risk flood areas are located in low-lying areas, whereas high-risk landslide areas are in mountains and hills with higher elevations and slopes), we mainly used population density, land-use type, distance to roads, and road density as evaluation indicators to solve this problem. Moreover, based on previous methods [27,31], three indicators (level of protection, construction date, and heritage type) were selected to evaluate the vulnerability of the heritage sites, hoping to provide references for follow-up studies.

### 5.2 Natural disaster risk priority by administrative districts

Although China has formulated a series of measures concerning the protection of cultural heritage, owing to insufficient financing and a lack of experience in cultural heritage disaster prevention, local cultural relics bureaus face practical difficulties and are seeking ways to efficiently protect and manage cultural heritage within a limited budget. This study fills the gap in natural disaster risk studies in Jinan and provides local governments with reliable information regarding the risk status of cultural heritage sites. Additionally, this information can help identify cultural heritage to which priority should be given in terms of protection, facilitate the early development of disaster prevention plans, and various forms of interventions to enhance the level of disaster prevention in areas where the cultural heritage sites are located, effectively addressing future natural disasters.

**Fig. 15:**
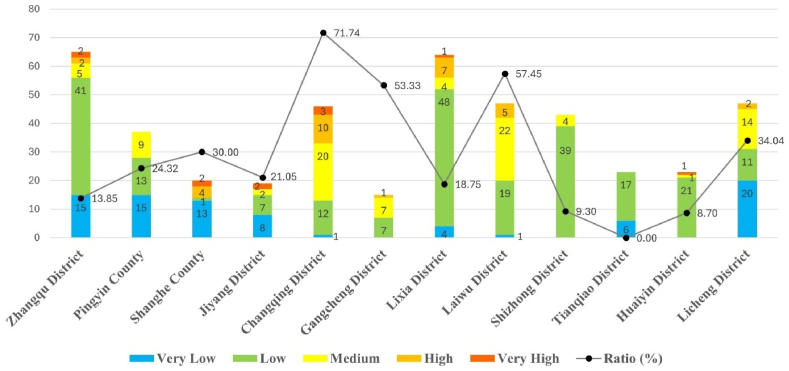
Risk priority by administrative districts.

Considering that city- and county-level cultural heritage sites that are more vulnerable to natural risks are typically managed by district/county governments and that conservation grants are allocated by the Jinan Municipal Government based on plans developed by each administrative district government, it is impossible to identify which administrative districts should be prioritized for support. This study counted the number of cultural heritage sites at various risk levels in 12 districts and counties of Jinan City and calculated the proportion of cultural heritage sites at medium and above risk levels. As shown in Fig. 15, the higher the proportion, the greater the risk to cultural heritage in the area. Therefore, it is necessary to develop timely measures to reduce the risk of natural disasters. Among them, Changqing District had the highest proportion. Based on the hazard maps of the four types of natural disasters, the western region is mainly threatened by flood disasters. Therefore, the construction of drainage facilities, such as diversion canals, could be considered to improve flood resilience. The central and eastern regions face potential hazards from the other three disaster types. In this regard, disaster risks can be controlled by strengthening structures, and attention should be paid to more vulnerable cultural heritage sites such as grottos and stone carvings. This type of heritage is mainly distributed in mountains and hills with high terrain and inconvenient transportation, thus necessitating local residents’ participation in protection and disaster prevention work and promptly addressing the risks of disaster occurrence. In addition, the proportion was over 50 % in Gangcheng District and Laiwu District. Therefore, Jinan City should prioritize improving disaster prevention and reducting cultural heritage in these three districts.

### 5.3 Limitations and future improvements

Although we adopted some reliable models and methods, the research results still have certain limitations owing to the limited data obtained. In flood hazard analysis, we used the SCS-CN method, which is less data-demanding, to predict flood hazard. However, this method, with a single and simplified runoff generation mechanism, is unable to analyze the impacts of some prolonged or persistent, very heavy rainfall events. Additionally, the study is based only on data such as PGA values for earthquake hazard prediction; although we refer to previous studies, due to fewer historical seismic records and lack of detailed geologic data, the analysis result is less reliable compared to that of the other three types of disasters. Therefore, further studies could acquire more accurate predictions by collecting more detailed data (e.g., observation data from rainfall stations, data on flood detention areas such as reservoirs and lakes in the watershed, and geological data) or using other types of models. Additionally, we have to admit that the four types of natural disasters mentioned above do not fully encompass the potential risks to cultural heritage. It is recommended that follow-up studies include other natural disasters that pose a greater threat to cultural heritage, such as wildfires, to provide a more comprehensive assessment.

Furthermore, there is no consensus in the existing literature on the best methodology or indicators for assessing multi-hazard risks to cultural heritage sites. Based on the UNESCO framework, this study selected representative indicators to measure the risks of natural disasters. However, more detailed data on cultural heritage sites and their surrounding environments enable a better reflection of their vulnerability to natural disasters. Thus, it is recommended, that in the future, departments such as the Cultural Relics Bureau publicly disclose data involving the materials, structures, and preservation status of each cultural heritage site and the density of the surrounding buildings.

## 6 Conclusions

Jinan City was selected as an example for this study. RS and GIS, combined with multiple methods and models, were used to analyze and evaluate the threats to cultural heritage from four types of natural disasters: floods, landslides, earthquakes, and soil erosion. A disaster risk map was drawn. The strength of this study lies in its use of methods and models such as SCS-CN, MaxEnt, and RUSLE, which allow for the objective and effective simulation of natural disaster impacts on cultural heritage sites. It not only identifies cultural heritage sites with high overall natural disaster risk but as well as the intensity of each hazard. This provides researchers and managers of cultural sites with a valuable reference to decide which sites need further detailed studies or disaster management plans.

The results of this study indicate that there are 130 cultural heritage sites in Jinan that are at moderate or high risk and that this number is likely to grow as the climate continues to change. Second, the risk of natural disasters is higher for cultural heritage sites at the city (county) level, especially for grottos, rock carvings, and ancient sites built before the Ming and Qing dynasties, where the risk is higher than for other cultural heritage sites. Finally, cultural heritage sites in three administrative districts (Changqing, Gangcheng, and Laiwu), had the highest risk priority.

Influenced by climate change and environmental damage, natural disasters such as heavy rains, floods, and landslides occur frequently and pose a significant threat to cultural heritage sites and their surroundings. The results of the study contribute to develop a more efficient analysis of the vulnerability of cultural heritage helpful for adaptation to climate change and eventually to insurance solutions [57,58]. It is hoped that the methodology and conclusions presented in this study will help cultural heritage protection departments and policymakers in Jinan select effective disaster prevention and mitigation countermeasures to meet the challenges posed by climate change, and efficiently protect and manage cultural heritage.

## Funding statement

The authors received no funding for this research.

## Additional information

No additional information is available for this paper.

## Ethics declarations

Review and/or approval by an ethics committee was not needed for this study because this study has no ethical implications.

## Data availability statement

Data included in article/supp. material/referenced in article.

## CRediT authorship contribution statement

**Guanyu Wei:** Writing – original draft, Visualization, Software, Methodology, Investigation, Data curation. **Gab-Soo Han:** Writing – review & editing, Visualization, Validation, Software, Methodology. **Xiaoxia Lang:** Writing – review & editing, Validation, Methodology, Investigation.

## Declaration of competing interest

The authors declare that they have no known competing financial interests or personal relationships that could have appeared to influence the work reported in this paper.
